# Qualitative evidence syntheses of attitudes and preferences to inform guidelines on infant feeding in the context of Ebola Virus Disease (EVD) transmission risk

**DOI:** 10.1371/journal.pntd.0010080

**Published:** 2022-03-10

**Authors:** Fiona Campbell, Andrew Booth, Christopher Carroll, Andrew Lee, Clare Relton

**Affiliations:** 1 School of Health and Related Research (ScHARR), University of Sheffield, Sheffield, United Kingdom; 2 Institute of Population Health Sciences, Queen Mary, University of London, London, United Kingdom; NIAID Integrated Research Facility, UNITED STATES

## Abstract

**Background:**

Breast-feeding holds considerable potential to reduce infant mortality. Feeding choices, already complex, take on additional complexity against a backdrop of the risk of transmissible Ebola Virus. This review describes the factors that influence infant feeding and attitudes of pregnant women, mothers, family members and health practitioners, policy makers and providers (midwives) concerning infant feeding when there is a risk of Mother-to-Child (MTC) transmission of Ebola Virus Disease (EVD).

**Methodology:**

A systematic review of qualitative studies identified through rigorous searches of thirteen online databases and additional citation searches of included studies was undertaken. Search terms included breast-feeding, breast-feeding, infant feeding; Ebola; and qualitative, interview(s) and findings. Independent extraction of data by two reviewers using predefined extraction forms. Studies were assessed using the CASP Qualitative checklist.

**Principal findings:**

5219 references were screened. 38 references related specifically to Ebola, and five papers met the inclusion criteria with data gathered from two settings: Guinea and Sierra Leone. The EVD outbreak had a significant impact on beliefs, attitudes, and resources to support infant feeding practices negatively affecting the nutritional status of children. The evidence from these studies highlight the need for guidance and appropriate psychosocial support need to be available to mothers who display symptoms and become infected and to front-line staff who are giving advice. Communities need to be engaged because stigma and fear may hinder uptake of appropriate interventions. The EVD outbreak caused multi-level system disruption akin to that seen following a natural disaster, meaning that logistics and coordination are critical and need adequate resourcing. Food production and distribution, and malnutrition screening are also disrupted and thereby compounding compromised nutritional status. The limited number of relevant studies highlights the need for further primary research, particularly in translation of messages to local settings.

**Conclusions:**

An EVD outbreak causes multi-level disruption that negatively impacts infant feeding and child care practices. Negative impacts have multiple causes and successful planning for Ebola outbreaks requires that nutrition of infants and young children is a priority. Lessons from the Ebola pandemic have wider applicability to other pandemic contexts including Covid-19.

## Author summary

An Ebola outbreak has been likened to a natural disaster in its devastating impact on child health and nutritional status. The ease of transmission of a disease with a 50% mortality rate for those once infected raises particular challenges for breast-feeding women. This systematic review explored the attitudes and preferences of pregnant women, mothers, family members, health practitioners and policy makers concerning infant feeding during an Ebola epidemic as documented in qualitative studies. Participants reported confusion and inconsistent guidance regarding breast-feeding practice, together with difficulties in conveying recommendations to separate mothers infected with Ebola from their breast-feeding infants. Disruption impacted on access to supplementary and complementary feeds, health systems monitoring child growth and support to child health. Widespread distrust and suspicion of health professionals hindered health care provision. Wider family assumed roles in feeding and caring for young children, within the context of reduced access to nutritious foods and supplementary feeds. The work of humanitarian agencies, while valuable, lacked coordination. Planning for an Ebola outbreak requires advance preparation of health messaging for mothers and caregivers, coordinated provision of complementary feeds and support for families who assume caring responsibilities. Strategies to ensure linked up working between government and humanitarian agencies are essential.

## Introduction

There are many infectious disease pathogens known to be transmissible from mother-to-child (MTC) such as Ebola Virus, Cytomegalovirus (CMV), Herpes Simplex Virus (HSV), the Hepatitis viruses, and Rubella virus (CDC Maternal and Infant Illnesses and Conditions). One of the recognized transmission routes is through breast-feeding, the main form of infant feeding in many countries worldwide but especially in low- and middle-income countries (LMICs). It is therefore important to understand those factors that influence decision-making around infant feeding in the context of transmissible illness, and to explore relevant stakeholders’ values and preferences around this issue, that is, their **beliefs, fears, perceptions and experiences**.

To this end, the WHO commissioned this qualitative evidence synthesis (QES) to help to understand the views of pregnant women, mothers, their families, health care workers and service providers concerning:

- Infant feeding options (breast-feeding, use of breastmilk substitutes or mixed feeding with both breastmilk and breastmilk substitutes) when either the mother or infant is suspected or confirmed to be infected with a potentially transmissible illness.
- The risks and outcomes of breast-feeding when either the mother or infant is suspected or confirmed to be infected with a potentially transmissible illness.
- Infant feeding options in areas of increased infectious disease transmission.

Substantive qualitative research has been conducted exploring the attitudes and barriers faced concerning infant feeding in the context of the risk of transmission of HIV/AIDS. However, no such systematic review or QES has been performed to date to understand the attitudes and preferences of pregnant women, mothers, their families, health care workers and service providers concerning infant feeding when there is the risk of transmission of Ebola Virus.

The focus of this report is therefore upon infant feeding within the context of an outbreak of Ebola Virus Disease (EVD). Unlike other infectious diseases, outbreaks of high consequence infectious diseases (HCIDs) such as EVD have considerable public health and societal impacts. HCIDs typically have high fatality rates, may lack effective detection and/or therapeutic measures, can spread rapidly in community and healthcare settings, and consequently require an enhanced population-wide system response. The consequences of outbreaks of HCIDs more closely resemble a disaster, such as the earthquakes in Nepal (2015) and Haiti (2010), due to the system level disruption that occurs. For example, the 2014–15 West African EVD outbreak disrupted entire food systems, losses in harvests, border closures, quarantines and other restriction measures disrupted the marketing of goods and agricultural produce [1].

As well as the unique level of disruption to health and food systems, difficulties in developing infant feeding policies and programmes in affected countries are exacerbated by uncertainty regarding the risk of transmission through breast-feeding for mothers who had survived EVD. The uncertainty about risks of transmission has led to inconsistent and changing breast-feeding recommendations during an Ebola outbreak [2]. Due to high mortality rate associated with EVD, family members may have to assume responsibility for orphaned infants. These distinctive features of Ebola outbreaks mean that it warrants a separate examination of the attitudes and preferences of populations targeted by guidelines.

### Qualitative evidence syntheses and evidence to decision frameworks

Given the complexity of health systems, it is challenging to identify appropriate evidence for determining policy choices. The challenge presented by EVD, requires the development of evidence-based health systems guidance, i.e. “systematically developed statements produced at global or national levels to assist decisions about appropriate options for addressing a health systems challenge in a range of settings and to assist with the implementation of these options and their monitoring and evaluation”[3].

The World Health Organization (WHO) has led the way in expanding the types of evidence used to develop health guidance beyond questions of effectiveness to encompass the attitudes and preferences of those affected by the guidance [4,5]. Evidence includes the attitudes and preferences of those receiving, delivering, planning and organising that care. Qualitative evidence syntheses (QESs) play a pivotal role in addressing such considerations. Qualitative research studies offer one mechanism for eliciting the attitudes and preferences of populations targeted by guidelines, to inform health policy and systems decisions. QESs gather studies from diverse contexts to explore what those affected by health guidance want, need and value [4–7]. They can inform both the broader aims of the guidance and also identify key outcomes to be considered when making recommendations [8]. The growing use of qualitative evidence to inform decision-making has been facilitated by several key developments in the field, including better standards for reporting primary qualitative studies [9], more robust methods for undertaking qualitative evidence syntheses [10], and databases for rapidly identifying primary studies within the health field [11]. Associated developments include the emergence of GRADE-CERQual—an approach for assessing how much confidence to place in findings from qualitative evidence syntheses [7–12], as well as frameworks to facilitate transparent and systematic assessment by decision makers [13]. GRADE-CERQual is the approach used to appraise the findings in this review.

Health decision making will be informed by a range of factors, including contextual factors such as resource availability. Evidence-to-decision (EtD) frameworks seek to ensure that all criteria of relevance to a health decision are systematically considered. They offer a structured approach for decision-making bodies to systematically consider the available evidence and to make judgements that are informed by a knowledge of the advantages and drawbacks of a given health decision].

Guidelines from the WHO provide recommendations for clinical practice, public health and health system strengthening, and are intended to support health decision-makers in prioritising or selecting suitable clinical, public health or health system interventions. The WHO formulates its recommendations using an EtD framework which encompasses eight criteria (see Table 1): quality of evidence, attitudes and preferences, balance of benefits and harms, resource implications, priority of the problem, equity and human rights, acceptability, and feasibility [14]. The ‘attitudes and preferences’ criterion is to be informed by the findings of this qualitative evidence synthesis.

**Table 1:** WHO Evidence-to-decision framework to determine the direction and strength of a recommendation.

Factor	How the factor influences the direction and strength of a recommendation
Quality of the evidence	The quality of the evidence across outcomes critical to decision-making will inform the strength of the recommendation. The higher the quality of the evidence, the greater the likelihood of a strong recommendation.
Attitudes and preferences	This describes the relative importance assigned to health outcomes by those affected by them; how such importance varies within and across populations; and whether this importance or variability is surrounded by uncertainty. The less uncertainty or variability there is about the attitudes and preferences of people experiencing the critical or important outcomes, the greater the likelihood of a strong recommendation.
Balance of benefits and harms	This requires an evaluation of the absolute effects of both benefits and harms (or downsides) of the intervention and their importance. The greater the net benefit or net harm associated with an intervention or exposure, the greater the likelihood of a strong recommendation in favour or against the intervention.
Resource implications	This pertains to how resource-intense an intervention is, whether it is cost–effective and whether it offers any incremental benefit. The more advantageous or clearly disadvantageous the resource implications are, the greater the likelihood of a strong recommendation either for or against the intervention.
Priority of the problem	The problem’s priority is determined by its importance and frequency (i.e. burden of disease, disease prevalence or baseline risk). The greater the importance of the problem, the greater the likelihood of a strong recommendation.
Equity and human rights	The greater the likelihood that the intervention will reduce inequities, improve equity or contribute to the realization of one or several human rights as defined under the international legal framework, the greater the likelihood of a strong recommendation.
Acceptability	The greater the acceptability of an option to all or most stakeholders, the greater the likelihood of a strong recommendation.
Feasibility	The greater the feasibility of an option from the standpoint of all or most stakeholders, the greater the likelihood of a strong recommendation.

### Eliciting attitudes and preferences from the literature

**Fig 1:**
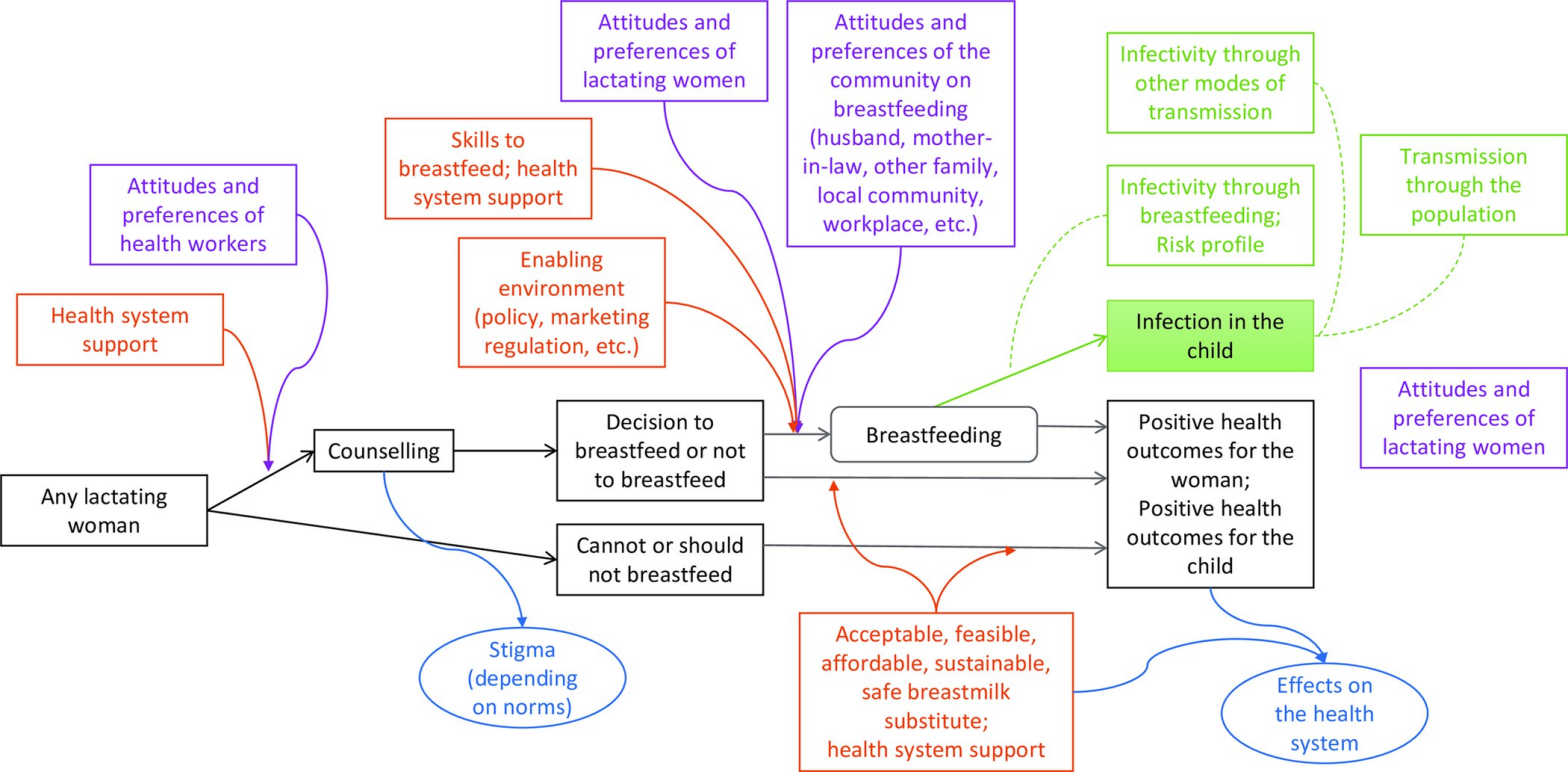
Analytic Framework produced by World Health Organization for Infant Feeding and Transmission of Infection, Rayco-Solon P 2019. [23]

In the context of the *Guideline*: *Infant Feeding in the Context of non-HIV Transmission Risk*, four specific types of attitudes and preferences were identified by the World Health Organization commissioning team for particular attention (Analytical Framework) (Fig 1):

1. Attitudes and Preferences of Health Workers in relation to Counselling of lactating women
2. Attitudes and Preferences of Lactating Women in relation to the decision to breastfeed or not breastfeed
3. Attitudes and Preferences of the Community (e.g. husbands, mothers-in-law, other family members, the workplace and the local community) in relation to support breast-feeding.
4. Attitudes and Preferences of Lactating Women in relation to the infection and possible transmission.

Undernutrition is associated with an estimated 2.7 million child deaths annually or 45% of all child deaths [15]. Infant feeding is key to improved child survival and promotes healthy growth and development. The first 2 years of a child’s life are particularly important, as optimal nutrition during this period lowers morbidity and mortality, reduces the risk of chronic disease, and fosters better development overall. It has been estimated that optimal breast-feeding will save the lives of more than 820,000 children under the age of 5 years each year and both the WHO and UNICEF(United Nations Children’s Fund) recommend initiation of breast-feeding within 1 hour of birth, exclusive breast-feeding for the first 6 months of life with introduction of complementary foods at 6 months with continued breast-feeding up to 2 years of age or beyond [16].

However, in the context of an infection there is a risk that the breast-feeding child may acquire infection through breast-feeding, e.g. ingestion of infected breastmilk as in the case of HIV. Alternatively, infection may occur through close contact with the mother, e.g. direct contact of infected bodily secretions such as tears (e.g. EVD), or airborne spread/inhalation of infected fomites (e.g. SARS, influenza, COVID-19). Current WHO guidelines recommend that asymptomatic breastfed infants of Ebola-infected mothers should be separated from their mothers and replacement fed (WHO 2015). However, the risk to breastfed babies and the contribution of breast-feeding to transmission is not fully understood [17].

Mothers who are considering breast-feeding face many challenges when making the decision whether to initiate or maintain this method of infant feeding. Pressures may exist at an individual, family or community level and in interactions with health service providers. An already complex decision may be compounded further when made against a backdrop of potential risk of disease transmission to the child. In such circumstances, the choice of infant feeding method extends beyond just the health benefits of breast-feeding, or the risks and advantages of different methods of infant feeding, and is likely to be heavily influenced by the attitudes and preferences of mothers.

Better understanding is required of the attitudes and preferences of pregnant women and mothers, together with other stakeholders who influence or are affected by decisions related to infant feeding (e.g. family members and health practitioners, policy makers and providers, in the context of the risk of disease transmission from breastmilk.

### Research question

What factors influence infant feeding attitudes and practices among healthcare workers, policymakers, and community members when the risk of contracting EVD via breastfeeding is present.

## Method

### Protocol and registration

This review was originally conceived as a review of “*acceptable medical reasons for use of breast-milk substitutes* in the context of transmissible disease.” The protocol was registered with the PROSPERO CRD database. It is published and available at PROSPERO 2019 CRD42019143387. Subsequently, the World Health Organisation asked the review team to focus on non-HIV transmissible infections [18], Zika virus [19] and Ebola virus. This review therefore represents the third of three related qualitative evidence syntheses following the same overall protocol.

### Inclusion criteria

To be included in the review and synthesis, studies were required to satisfy the following criteria, defined using the PerSPEcTiF(S) framework [20] (Table 2).

**Table 2:** Inclusion criteria, defined using the PerSPEcTiF(S) framework [20].

Perspective(s)	Women, partners, carers and significant others, healthcare providers, policy makers
Setting	Any setting (primarily community settings)
Phenomenon of interest	Infant feeding in the context of the risk of transmission of Ebola
Environment	International, particularly Low- and Middle-Income countries (LMICs) where transmissible diseases are more prevalent
Comparison	*[Implicitly compared with attitudes and preferences concerning infant feeding where there is no transmission risk]*
Timing	When contemplating, carrying-out or supporting breast-feeding, breast milk feeding or alternative infant feeding
Findings	Attitudes and preferences: fears, perceptions, experiences and beliefs regarding the phenomenon of interest
Study Design	Qualitative studies. Surveys with qualitative data as free text responses to survey questions were excluded

### Information sources and search strategy

The following databases were searched to identify relevant published and unpublished literature from 2000 to 2019:

- CINAHL (Ovid);
- MEDLINE (Ovid);
- EMBASE
- PsycINFO (Ovid);
- Social Science Citation Index (Web of Science);
- POPLINE;
- LILACS
- African Journals Online

There was no restriction by publication type. Non-English language studies were included where a translation was available or was possible. Using guidelines developed by the Cochrane QIMG for searching for qualitative evidence [21], search strategies were developed for each database. The search combined thesaurus and free-text terms for an extensive list of transmissible diseases (see above), with terms for infant feeding, breast-feeding and breast milk, and published filters to identify qualitative research [11,22]. No geographic restrictions were imposed on the search. The date range was limited to 2000–2019 to capture the most recent and contemporary views expressed by relevant people on this topic. The reference lists of all the included studies and the key publications (i.e. any relevant systematic reviews) were all searched. In addition, a citation search was performed on Google Scholar for key included articles. Citation updates were set up on Google Scholar for all included studies to identify newly published studies published up until the final analyses and further studies retrieved for inclusion. An update search was conducted from 2019 to 2020, following publication of the WHO report and no further studies were identified.

### Study selection, extraction and appraisal

Using the inclusion criteria, preliminary study screening of all titles and abstracts was conducted by one reviewer (AB) to identify potentially relevant papers, and full text screening of the results was conducted independently by at least two reviewers (FC, AB or CC). A data extraction form was developed and piloted by two reviewers. Two reviewers (CC, FC) then independently performed data extraction and quality assessment (using the CASP Checklist: *10 questions to help you make sense of a Qualitative research*) of all included studies using the agreed form; any inconsistencies were resolved by discussion and, if necessary, consultation with a third reviewer (AB). Screening of the updated search was undertaken by one reviewer (FC).

### Synthesis

The synthesis used relevant domains from a WHO/UNICEF conceptual model (Fig 1) for HIV and infant feeding as a theoretical framework to categorise and organise the relevant data [23]. This model depicts conditions that influence feeding decisions and their outcome (e.g., knowledge, perceptions, family influences, resources, environment, etc), and issues to be explored in order to define appropriate feeding options. This framework depicts an ecological model, which assumes that feeding behaviours are influenced by interacting, intrapersonal, social and cultural, and physical environment variables. The key concepts in the developed *a priori* framework were as follows:

- Factors relating to the individual;
- Family and community-related factors;
- Health system factors;
- Socio-economic factors

In line with Cochrane methodological guidance, it is not considered necessary for a best fit framework to correspond exactly to the phenomenon of interest [10]. The salient features were considered to be the context of infant feeding and the presence of a transmissible infection—in the case of the model, HIV, and in the case of this review, Ebola virus.

## Results

**Fig 2:**
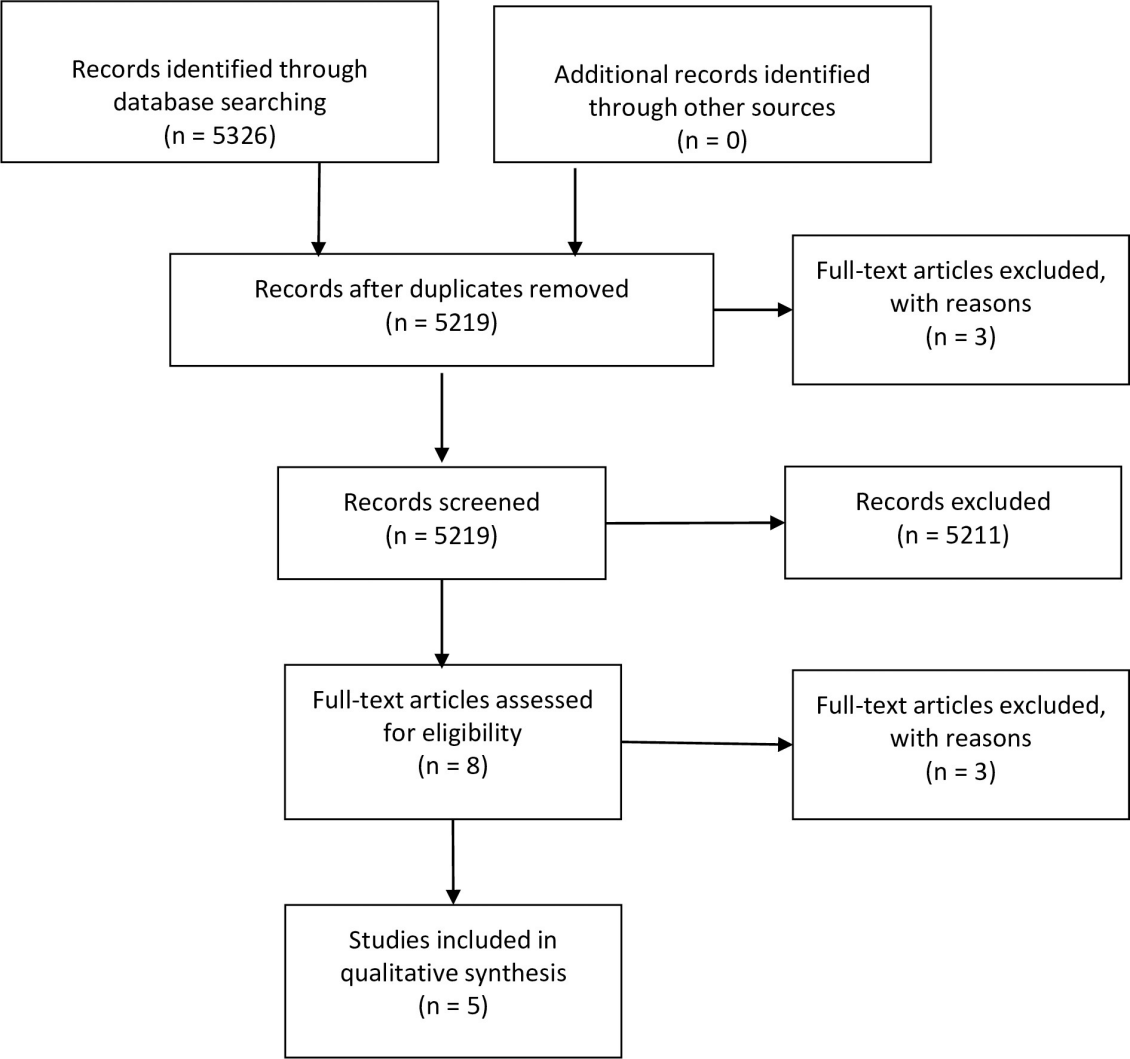
PRISMA flow diagram.

Details of the study selection process are presented in Fig 2. The search retrieved 5219 potentially relevant relating to a variety of infectious diseases. Thirty-eight references were specifically related to Ebola Virus Disease. Of these, 28 papers were excluded at title and abstract stage, while a further 4 papers were excluded at the full text stage. The reasons for exclusion at full text included: duplicate publication [24] and a lack of qualitative evidence on infant feeding and infection transmission risk [25–27].

Six publications (5 studies) satisfied the inclusion criteria (see Table 1). All five studies were conducted in African countries: Guinea [28], Sierra Leone [29–32] or Guinea and Sierra Leone jointly [33]. Three Ebola studies, undertaken by the same team, explored the views of survivors, health providers, managers, government officials, civil servants and staff from international agencies [28,29]. Kodish et al (2019a) [33] aimed to garner stakeholder perspectives and build consensus around the key nutrition related challenges faced, as well as the important lessons to inform strategies for addressing nutrition in future outbreaks, drawing participants from both Guinea and Sierra Leone and triangulating these results from those gained in the earlier studies [28,29]. Elston et al (2017) [31] used qualitative and quantitative methods to understand and describe the impact of the Ebola virus and consequent health needs. They included interviews across two districts in Sierra Leone with key local stakeholders, community members and mother’s with young children. McMahon et al (2016) [32] conducted interviews with health providers working in community health settings and child health centres across two districts to examine how the Ebola epidemic changed their professional, personal and social lives. The five studies represented a partial match to the focus of this review, given their focus on nutrition more broadly compared in the specific context for this review which is infant nutrition. We have therefore only drawn upon that subset of data that is relevant to infant feeding. The characteristics of the five included studies are reported in Table 3.

**Table 3:** Characteristics of included studies.

Author (Date)	Country	Vicinity (i.e. Region, State, Province, City)	The study’s aims and purpose?	Perspectives and sample characteristics	How was the sample selected?	Data collection methods used?
Elston 2016 [30]	Sierra Leone	Sierra Leone–in two districts, on in the Southern Region and one in the Northern Region	A mixed methods approach to understanding and describing the impact of the Ebola virus and consequent health needs.	60 interviews were conducted in total across both districts with participants including: key local stakeholders (District Health Management Teams (DHMT), members of Ebola response teams; civil and traditional authority,health care workers, community workers, social mobilizers, patients and members of non-governmental organizations (NGOs). Six focus groups were also conducted with: community members, a women’s group, mothers with children attending a child health clinic, social mobilizers and town council members.	unclear	Mixed methods were used, but little detail of the qualitative component. Interviews and focus groups. Thematic analysis was performed to identify common themes.
Kodish (2018)[28]	Guinea	Not reported: From 5 of Guinea’s 8 administrative regions that were most impacted by Ebola	To understand how Ebola outbreak may have impacted infant and young child nutrition in Guinea. Second, to understand how stakeholders at multiple different levels perceived the acceptability and effectiveness of the nutrition-specific response during this outbreak to draw lessons learned and make recommendations for consideration in future similar scenarios	Community, Midwives, Health Providers, Health Managers/Decision-makers, Government Officials/Civil Servants, International Organisations or Agencies n = 27: 11 key informants represented perspectives from bodies including Government/Policy, United Nations, Hospital Management, and NGOs. 16 individual perspectives (7 front-line health workers, 6 household/community members of Ebola victims, 2 community leaders, and 1 Ebola survivor). Individuals representing both community and professional roles participated from across 5 of Guinea’s 8 administrative regions most impacted by Ebola.	Purposive	Interviews
Kodish (2019a) [33]	Guinea and Sierra Leone	Not reported	To generate multiple stakeholder perspectives for understanding the nutrition challenges faced during the Ebola virus disease outbreak, as well as for consensus building around improved response strategies	Government Officials/Civil Servants, International Organisations or Agencies n = 36 (17 from Guinea, 19 from Sierra Leone [including 4 Ebola survivors]).	Purposive	Interviews, Participatory workshops
Kodish (2019b) [29]	Sierra Leone	Across all four provinces	To explore how and through what pathways EVD outbreak impacted nutrition in Sierra Leone. To investigate factors to effective implementation of nutrition response strategies during EVD outbreak. To use findings to consider a nutrition preparedness and response framework in planning for future outbreaks.	Phase 1: Government hospital managers, government policy makers. Managers working with NGOs or United Nations organisations involved in the outbreak response at the national level. Phase 2: EVD survivors, community leaders, health workers	Purposive sampling based on role and geographic representation.	Semi-structured interviews
McMahon (2016) [32]	Sierra Leone	Across two districts during the 2014–15 Ebola epidemic	To examine how front-line providers in Sierra Leone experienced and assessed changes in their professional, personal and social lives during the protracted Ebola outbreak	35 health care providers from eight peripheral health units (PHUs). The PHUs were government-run and included Community Health Posts, Maternal and Child Health Posts and Community Health Centres.	All providers from included units Unclear how selected	semi-structured interviews open inductive coding informed by grounded theory Coding shared and validated

**Fig 3:**
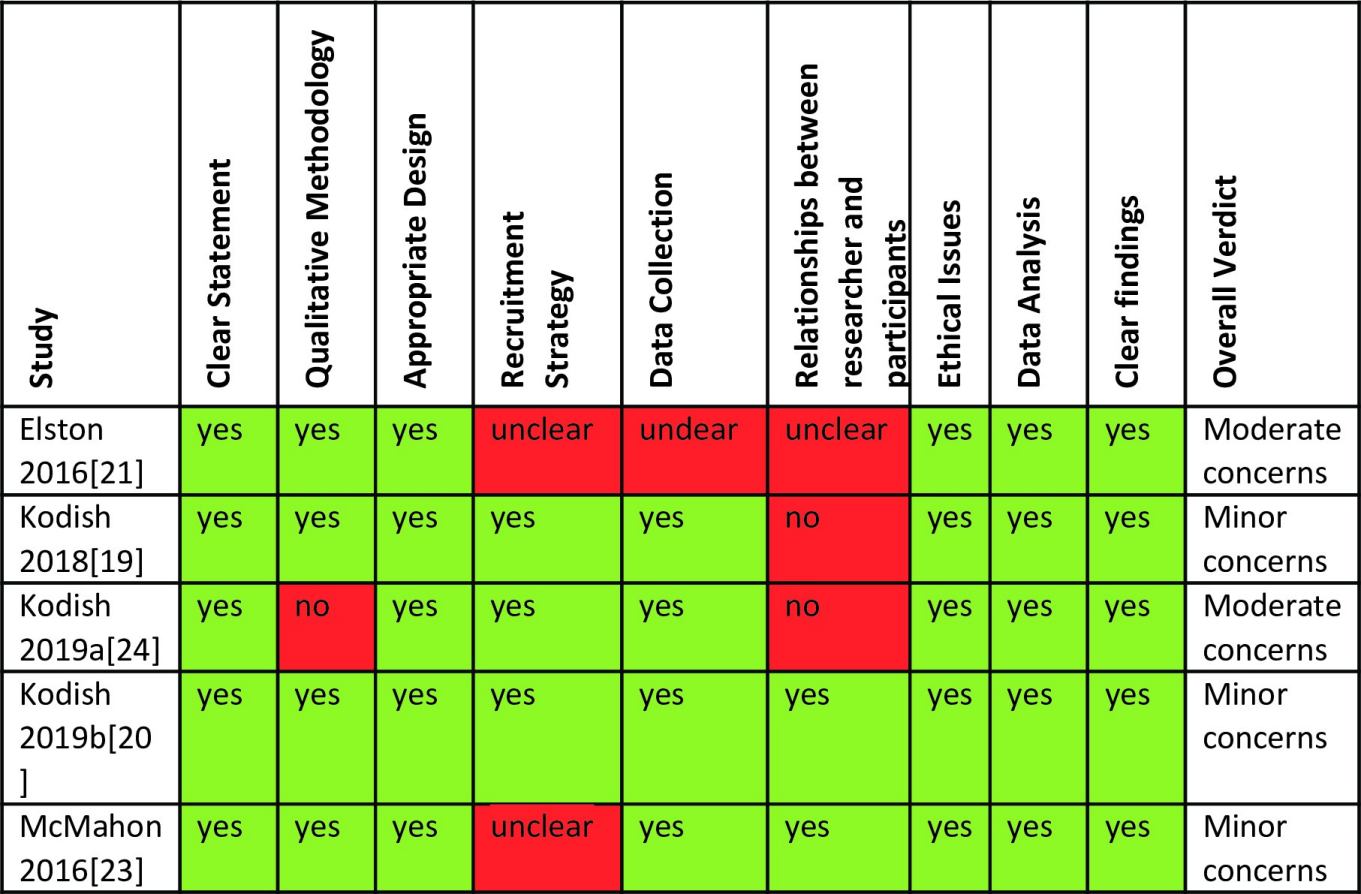
CASP quality assessments for studies on attitudes and preferences concerning infant feeding in the context of Ebola virus disease transmission risk.

Quality assessment of all five studies using the CASP checklist revealed that characteristics of the evidence base do not pose a substantive risk to rigour (see Fig 3). Four of the studies presented a clear question, qualitative design, use of appropriate methodology, and reporting clear findings. A mixed methods study [30] did not report the qualitative methods is sufficient detail to judge all quality domains. The studies also clearly reported rigorous recruitment and data collection and analysis strategies. One study adequately addressed the relationships between researchers and participants (reflexivity) [29]. Methodological limitations of all five studies were further explored as part of the GRADE-CERQual assessement (see Table 4).

**Table 4:** GRADE-CERQual Summary of Qualitative Findings.

Summary of review finding	Studies contributing to the review finding	Methodological limitations	Coherence	Adequacy	Relevance	GRADE-CERQual assessment of confidence in the evidence	Explanation of GRADE-CERQual assessment
Individual level factors							
Front line workers were not always able to provide guidance and information on breast-feeding for mothers with symptoms of Ebola, confirmed infection and following an infection in a timely or a consistent manner.	Kodish 2018, 2019b [28,29]	Two studies, one with some methodological limitations due to limited sample sizes that did not allow data saturation but did allow for identification of salient themes.	Well supported finding, though the voices of key stakeholders, including mothers who did not survive are missing.	Minor concerns about adequacy due to limited number of studies and limited sample size	as studies report on two outbreaks in two countries only. Studies not focused on infant and child health but on nutrition more broadly	High confidence	Two studies (Sierra Leone, Guinea) with few methodological limitations and rich description.
Health professionals find it challenging and difficult to advise mothers with Ebola to cease breast-feeding and to manage the separation of mothers from infants within a context where exclusive breast-feeding is widely advocated and adopted.	McMahon et al 2016 [32] Kodish et al 2018 [28], 2019b [29]	Three studies, one with some methodological limitations due to limited sample sizes that did not allow data saturation but did allow for identification of salient themes.	Well supported finding, though the voices of key stakeholders, including mothers who did not survive are missing.	Minor concerns about adequacy due to limited number of studies and limited sample size	Partial relevance as studies report on two outbreaks in two countries only. Studies were not focused on infant and child health but nutrition more broadly	High confidence	Two studies (Sierra Leone, Guinea) with few methodological limitations and rich description.
Community level factors							
Fear and stigma during an EVD outbreak may result in misleading beliefs, altered health seeking behaviour and limited acceptability of messages	McMahon 3016 [32] Elston 2016 [30], McMahon 2016 [32] Kodish 2018 [28], 2019b [29]	Four studies, one with minor methodological limitations due to limited sample sizes that did not allow data saturation but did allow for identification of salient themes.	Well supported finding, though the voices of key stakeholders, including mothers who did not survive are missing.	Minor concerns about adequacy due to limited number of studies and limited sample size	Partial relevance as these studies report on two outbreaks in two countries only. Studies not focused on infant and child health but on nutrition more broadly	High confidence	Two studies (Sierra Leone, Guinea) with few methodological limitations and rich description.
Extended families and communities become care givers for infants and young children who are orphaned or separated from their primary care giver and assume responsibility for their nutrition	Kodish 2018 [28], 2019b [29]	Two studies, one with minor methodological limitations due to limited sample sizes that did not allow data saturation but did allow for identification of salient themes.	Well supported finding, though the voices of key stakeholders, including mothers who did not survive are missing.	Some concerns about adequacy due to sample size	Partial relevance as studies report on two outbreaks in two countries only. Studies not focused on infant and child health but on nutrition more broadly	High confidence	Two studies (Sierra Leone, Guinea) with few methodological limitations and rich description.
Frontline worker, managers and policy makers must plan respectful responses that engage and involve the community. This is critical to the success of interventions.	Elston 2016 [30], Kodish 2018 [28], 2019b [29], 2019a [33]	Four studies, two with minor methodological limitations due to limited sample sizes that did not allow data saturation, and absence of a follow-up phase allowing dissenting viewpoints to be explored.	Well supported finding, though the voices of key stakeholders, including mothers who did not survive are missing.	Some concerns about adequacy due to sample size and lack of follow-up	Partial relevance as studies report on two outbreaks in two countries only. Studies not focused on infant and child health but on nutrition more broadly	High confidence	Four studies (Sierra Leone, Guinea) with few methodological limitations and rich description.
Socio-economic factors							
Policy makers and managers in preparing for an EVD outbreak must anticipate that R ready to use infant formula and nutritious complementary foods will become less accessible due to cost and disruption to transport and quarantine arrangements.		Two studies, one with minor methodological limitations due to limited sample sizes that did not allow data saturation but did allow for identification of salient themes.	Well supported finding, though the voices of key stakeholders, including mothers who did not survive are missing.	Some concerns about adequacy due to sample size	Partial relevanceas these studies report on two outbreaks in two countries only. Studies were not focused on infant and child health but nutrition more broadly	High confidence	Two studies (Sierra Leone, Guinea) with few methodological limitations and rich description.
Health System Factors							
Health systems facing an Ebola outbreak encounter a similar impact to a major disaster disrupting entire nutrition and health systems and programmes, altering care practices for infant and young children. Preparedness is critical to ensuring appropriate responses and with a high priority placed on nutrition.	Elston 2015 [30], Kodish 2018 [28], 2019b [29], 2019a [33]	Four studies, two with minor methodological limitations due to limited sample sizes that did not allow data saturation, and absence of a follow-up phase allowing dissenting viewpoints to be explored.	Well supported finding, though the voices of key stakeholders, including mothers who did not survive are missing.	Some concerns about adequacy due to sample size and lack of follow-up	Partial relevanceas these studies report on two outbreaks in two countries only. Studies were not focused on infant and child health but nutrition more broadly	High confidence	Four studies (Sierra Leone, Guinea) These are studies with few methodological limitations and rich description.
Stakeholders face coordination complexity and additional logistic challenges created by the arrival of new international organisations during the EVD outbreak.	Kodish 2018 [28], 2019b [29], 2019a [33]	Three studies, two with minor methodological limitations due to limited sample sizes that did not allow data saturation, and absence of a follow-up phase allowing dissenting viewpoints to be explored.	Well supported finding, though the voices of key stakeholders, including mothers who did not survive are missing.	Some concerns about adequacy due to sample size and lack of follow-up	Partial relevance as these studies report on two outbreaks in two countries only. Studies were not focused on infant and child health but nutrition more broadly	High confidence	Three studies (Sierra Leone, Guinea) These are studies with few methodological limitations and rich description.
Effective communication delivered in multiple languages, via diverse media could lead to positive changes in IYC feeding	Kodish 2018 [28], 2019b [29], 2019a [33]	Three studies, two with minor methodological limitations due to limited sample sizes that did not allow data saturation, and absence of a follow-up phase allowing dissenting viewpoints to be explored.	Well supported finding, though the voices of key stakeholders, including mothers who did not survive are missing.	Some concerns about adequacy due to sample size and lack of follow-up	Partial relevanceas these studies report on two outbreaks in two countries only. Studies were not focused on infant and child health but nutrition more broadly	High confidence	Three studies (Sierra Leone, Guinea) These are studies with few methodological limitations and rich description.

The findings of the synthesis, with evidence-based themes presented under the key concepts derived from the World Health Organization (2004) *a priori* framework for organising the evidence, are presented below in both narrative and tabular forms

### Individual level factors

Poor infant and young child (IYC) feeding and care giving practices and changing breast feeding practices were ranked amongst the most important challenges during the EVD outbreak in both Guinea and Sierra Leone [33].

Repondents in Sierra Leone reported that they were waned that if they felt unwell to not touch their child, or to feed them and also if one had been infected with Ebola but survived, were advised to no longer engage in exclusive breast-feeding [33]. One study describes how, during the EVD outbreak in Sierra Leone, infants were separated from their mothers to prevent vertical transmission [29]. Separating infants from infected breast feeding mothers was a culturally difficult guideline to apply [28]. Distrust and scepticism toward the health system and health workers hindered uptake of advice [30,32]. These factors all contributed to changes and deterioration in child and infant nutrition during the EVD outbreak.

Uncertainty, inconsistency in guidelines and poor implementation of guidance characterised advice to breast-feeding mothers during the Ebola outbreak in Sierra Leone and Guinea. Front-line health workers lacked knowledge and clear and consistent nutrition guidelines to draw upon when advising mothers about infant feeding if they were infected or in the aftermath of an infection. Health workers also expressed concerns about providing contradictory guidance: normally they would promote exclusive breast feeding, but in the context of an EVD outbreak they had to issue diametrically opposite advice to recommend that breast-feeding ceased and infant formula be used [28,29,33].

Front line workers in Sierra Leone and Guinea were not always able to provide guidance and information on breast-feeding for mothers with symptoms of Ebola, confirmed infection and following an infection in a timely or a consistent manner.

Front line workers faced challenges modifying breast-feeding, complementary feeding and caregiving practices. There was uncertainty and lack of clear guidance on when, and for how long, a mother with symptoms should stop breast-feeding, and when breast-feeding could recommence. In addition, there were challenges in access to ready-to-use milk and complementary foods, as well as mothers’ adherence to traditional practices regarding weaning foods that were resistant to change and exacerbated the risk of malnutrition. Health professionals find it challenging and difficult to advise mothers with Ebola to cease breast-feeding and to manage the separation of mothers from infants within a context where exclusive breast-feeding is widely advocated and adopted. There was stigma within communities, associated with stopping breast-feeding in an environment where exclusive breast-feeding is the norm and strongly advocated. Front line health workers expressed the dilemma when they had to change their message on exclusive breast-feeding [28,29]. In Sierra Leone it became more socially acceptable to stop breastfeeding if the mother or child were infected following more coordinated and enhanced awareness raising.

A notable gap in the data is the perspective of mothers, particularly in the context of the practice of separating a mother from her infant and what information and support they needed. Very limited evidence is available to explore mothers’ attitudes and preferences or the attitudes and preferences of those who did not survive.

### Family and community related factors

Increasing numbers of orphaned children and the temporary absence of primary caregivers means the wider and extended family [25] having to assume responsibility for feeding and caring for young children. This resulted in multiple challenges within a community where availability of, and access to, nutritious foods was already compromised [29] and this adversely affected caregiving.

Fear and mistrust were further factors during the EVD outbreak, for example, in Guinea the high mortality rates led people to believe that replacement milks for children who could no longer breastfeed and nutritious complementary foods given out by health staff contained Ebola [28]. Delays and reductions in health seeking behaviour and health facility attendance [30] disrupted health systems further and hindered the identification of cases of malnutrition [29]. These factors all worsened the nutritional and health status of infants and young children.

One of the key lessons reported from the experience in Sierra Leone was the need to put in place surveillance systems in order to identify malnutrition cases [33]. Another insight was the importance of working with communities for successful interventions and for training to occur at community level to tackle erroneous local perceptions [28]. Early denial of the EVD outbreak by community members and reduced health seeking behaviours were examples of community level factors that exacerbated the severity of the outbreak [29]. Stigma and the influence of socially acceptable behaviours also drove feeding practices with infants initially becoming infected as it was seen as socially unacceptable to stop breast-feeding [29].

The success of interventions addressing infant feeding practices was influenced by the community acceptability of the interventions. Appropriate social and behaviour change communication was essential and initial attempts from Government, United Nations and NGOs led to confusion and unanticipated reactions. In Guinea, initial messages were misunderstood at a community level and led to reduced care seeking, for example, messages to seek care were not responded as it seemed to contradict the message that the disease was incurable. This meant involving the community in designing these strategies, using multiple means of communicating messages and providing training at a community level. Greater coordination of messaging, and pilot testing were important in message design. The messenger was important, with greater confidence and trust placed in interpersonal communication from respected and known individuals such as community leaders, and health workers. Perceptions of the trustworthiness of health workers and the health system were also undermined influenced by rumours and misinformation about their role in the spread of EVD [30]. Sustained engagement with communities was essential. Community level committees also were valuable in liaising between the biomedical and local communities [28]. The need to convey nutrition related information leading to social and behaviour change, and the value of using multiple channels of communication such as print and mass media as well as interpersonal and ensure information was culturally appropriate and understandable was also a lesson learned in Sierra Leone during the pandemic [29].

### Health system factors

The initial stages of the EVD epidemic were marked by a lack of coordination of humanitarian efforts, poor dissemination of public health information, and a failure to prioritize IYC nutrition, but many of these factors improved over time. The authors noted that nutrition was not prioritised in the policy response to the EVD outbreak. In both Sierra Leone and Guinnea political will and policy were identified as one of the most important factors in responding to nutritional needs. ALack of political commitment to addressing nutrition during the EVD outbreak led to poor IYC feeding practices during the EVD outbreak [28]. In addition, during the EVD outbreak, health systems were severely disrupted, with household and community quarantines reducing movement [32]. Initially, the community experienced poor understanding of when to access health care [29]. Staff working for organizations, including government and non-government sectors in SL described that the focus of attention by intervening organisations was on EVD containment and treatment activities, and in this context nutrition activities including screening malnutrition cases was more difficult. Screening for malnutrition in children was further worsened by travel restrictions, and made it harder for people to seek help.

The arrival of new international organizations responding to the crisis contributed another layer of complexity to planning and coordination at the organisational level. During the initial stages of the outbreak there was the lack of information. Information was poorly disseminated and poorly coordinated by government agencies, health providers and voluntary organisations. This improved during the course of the outbreak. Behaviours were successfully changed and led to sustained improvements in child feeding practices.

Previously existing nutrition programmes, along with other major health programmes were adversely affected by the epidemic [30]. The Ebola outbreak weakened household food security that led to changes in complementary feeding practices, including the use of more affordable but less nutritious food substitutes, and a reduction in food quantity. The provision of milk substitutes and resources by humanitarian organizations and donors were regarded as a facilitating factor that improved response efforts. Food assistance was highly accepted and much needed. Front-line workers found that although children used to breast-feeding took some time to adjust to ready-to-use infant formulas, most did so without too much trouble [29]. Household food insecurity could be addressed through better system wide planning for food distribution. Indeed, the development of district level working committees and careful planning of food distribution were factors that improved coordination and logistics during the outbreak.

### Socio-economic factors

It is clear from all of the included studies [28–30,32,33] that the impact of the EVD outbreak and its resulting effects on infant nutrition were exacerbated because these countries are amongst the poorest in the world. The Ebola outbreak in Guinea and Sierra Leone led to increased levels of household food insecurity, which contributed to changes in IYC feeding practices. Many households lacked the ability to access nutritious complementary foods and mobility restrictions put in place meant others were unable to help [28]. The EVD outbreak exacerbated economic hardship and further reduced capacity to access much needed nutrition [28,29]. In Sierra Leone, even before the outbreak of EVD, the infant and young child nutrition was poor, with only 32% of children under 6 months exclusively breastfed and high levels of stunting and wasting. Rural communities were at even greater risk in Sierra Leone due to difficulties in delivering food assistance [29].

The EVD outbreak in low-income economies, with a GNI per capita of $1,045 or less), exposed the vulnerably of an under resourced health system and weak surveillance and administrative infrastructure. These led to reduced access to health care, health service utilization and therefore greater nutrition-related morbidity and mortality. The authors highlight the need for national level resources, to strengthen institutional capacity, enabling effective community based nutritional programmes that can protect newborn and child health [33].

## Discussion

The five studies [28–30,32,33] in the review reveal, that an Ebola epidemic is a disaster that causes multi-level disruption, weakening health systems, and livelihoods. The readiness of transmission coupled with a 50% mortality rate causes widespread fear, breeding distrust and disintegration of social support networks and communities. The breast fed infant is particularly vulnerable where risk of transmission is present.

In the Ebola outbreak, messages to mothers who were breast-feeding and frontline workers were not clear, timely or consistent. Messages to stop breast-feeding to prevent vertical transmission and the separation of mothers from infants were challenging within a culture where exclusive breast-feeding is the norm and widely advocated for child health. Caregivers themselves were uncomfortable separating a breast-feeding mother from her child.

However, health messages and advice for mothers was often unclear and poorly conveyed. Fear and stigma also influenced health behaviours and beliefs. In addition, the affordability and accessibility of alternative food was a major issue. Access to formula milks for infants separated from their mothers was hampered by their high cost, the diminished economic status of families, and poor access with food supplies disrupted. The high mortality rate and the needs to protect children from vertical transmission meant that the extended family had to assume caring roles for infants and young children. They too had reduced access to formula milks and nutritious supplementary foods. There is also the risk of diarrhoeal diseases in babies fed formula milk made with unsafe water in areas where access to clean water or the means to make them safe is limited. However, during the EVD epidemic, access to food supplements, milk replacements and hygienic feed preparation was also disrupted. Consequently, the provision of food assistance in food insecure areas is vital.

### Strengths and limitations

The five studies [28–30,32,33] were of high methodological quality, but the evidence base is limited by a lack of qualitative research. It was valuable that data was collected from two separate settings and further work is needed to explore different contexts, for example areas with less widespread infection. Most notably there was limited reporting of the attitudes and preferences of mothers themselves and as data was gathered retrospectively it therefore excludes those who did not survive. Further research is therefore warranted to explore particularly the views and experiences of mothers and their support needs, and to explore the impact of EVD outbreaks in different settings. The importance of attitudes to breast milk substitutes and how these may vary and be influenced by health messages are also questions that warrant further research.

### Conclusion

Nutrition advice needs to be prioritised and incorporated into advance planning at all levels of the health system, from local to national, with adequate resource and staff to implement plans. In the context of public fears and anxieties around the safety of infant feeding practices, the clarity and efficacy of public health communication as well as community engagement are vital. Agencies responding to nutrition crises during an EVD outbreak therefore need to deliver community level actions as well as consider the need for the provision of psychosocial support. Enhanced coordination between government and non-governmental actors, advance preparation of standard operating procedures, guidelines and better nutrition monitoring, all help improve the nutrition response during an EVD outbreak and protect the most vulnerable, the infant.
